# Effect of Solidification Direction on the Freckle Formation in Single-Crystal Superalloy Castings

**DOI:** 10.3390/ma18071534

**Published:** 2025-03-28

**Authors:** Dexin Ma, Hongyuan Sun, Yunxing Zhao, Weitai Xu, Zaiwang Huang, Bowen Cheng, Yang Liu, Fu Wang, Qiang Yang, Lv Li, Yangpi Deng, Fuze Xu, Haijie Zhang, Menghuai Wu

**Affiliations:** 1 State Key Laboratory for Powder Metallurgy, Central South University, Changsha 410083, China; sunhy2018@csu.edu.cn (H.S.); zhaoyunxing@csu.edu.cn (Y.Z.); huangzaiwang@csu.edu.cn (Z.H.); 2 Wedge Central South Research Institute, Co., Ltd., Shenzhen 518045, China; 3 Shenzhen Wedge Aviation Technology Co., Ltd., Shenzhen 518045, China; 18706102306@126.com (W.X.); cbwlsxx@sina.com (B.C.); lilv000@163.com (L.L.); dengyangpi030@163.com (Y.D.); xufuze12@163.com (F.X.); 4 State Key Laboratory for Manufacturing System Engineering, Xi’an Jiaotong University, Xi’an 710049, China; leoyounglab@163.com (Y.L.); fuwang@xjtu.edu.cn (F.W.); qiangyang@xjtu.edu.cn (Q.Y.); 5 Chair for Simulation and Modelling of Metallurgy Processes, Department of Metallurgy, University of Leoben, 8700 Leoben, Austria; haijie.zhang@unileoben.ac.at (H.Z.); menghuai.wu@unileoben.ac.at (M.W.)

**Keywords:** superalloy, single crystal, freckle, solidification direction, solutal convection

## Abstract

Solidification experiments in two opposite directions were conducted to investigate the buoyancy effect on freckle formation during directional solidification in single-crystal superalloy castings. During conventional upward solidification with the superalloy CMSX-4, severe freckles were observed in castings of various geometries. By reversing the solidification direction from upward to downward, freckle-free castings could be obtained. To visually verify the effect of the solidification direction, an in situ observation experiment by varying the solidification direction was performed using a Ga-In alloy. In the upward solidification process, strong solutal convection was visually observed due to the decrease in the density of the interdendritic liquid. Conversely, a stable condition without visible flow was established during downward solidification, due to the stable state of the top-light, bottom-heavy liquid system. A new Rayleigh-number model was successfully applied to characterize the freckle features in superalloy cluster castings. When the solidification direction was reversed from upward to downward, the driving force for solutal convection was suppressed, leading to the complete elimination of freckle formation in single-crystal superalloy castings.

## 1. Introduction

Due to the excellent high-temperature mechanical properties and corrosion resistance, nickel-based superalloys have been the optimal material for producing key high-temperature components in aero-engines and industrial gas turbines, such as turbine blades [1,2,3,4]. The global market for nickel-based superalloys in aero-engines and industrial gas turbine applications is projected to exceed USD 6.5 billion by 2030, driven by their critical role in high-temperature components such as turbine blades, which account for over 40% of the material demand in gas turbine manufacturing [5]. Single-crystal (SC) turbine blades manufactured through directional solidification processes are extensively utilized to enhance the efficiency and performance of turbine blades. The absence of grain boundaries in SC blades eliminates potential sites for stress concentration and failure, thereby significantly improving their mechanical properties and durability under extreme operating conditions [6,7,8,9].

In conventional production processes (Bridgman process) for SC superalloy turbine blades, the ceramic mold shell was filled with overheated superalloy melt and lowered from the heat chamber, through an isolation baffle, into the cooling zone. During this process, upward dendritic solidification occurs from the bottom to the top of the castings, counteracting gravity. Due to the segregation of alloying elements, heavy elements like W and Re concentrate in the dendritic solid phase (γ phase), while light elements such as Al and Ti are rejected into the residual liquid between dendrites. This results in a significant decrease in liquid density in the mushy zone compared to the overlaying bulk liquid. Under the gravity effect, this up-heavy and bottom-light state is unstable, and a strong convective flow from the mushy zone into the buck liquid is unavoidable. This causes remelting and fracturing of the dendrite arms, ultimately resulting in the vertical chain-like distribution of freckles defects [10,11,12,13,14,15,16,17,18,19]. Since the freckles contain high-angle boundaries transversal to the withdrawal direction, they have a detrimental effect on the creep rupture life and fatigue resistance of the material.

Freckles are presently one of the primary cast defects in the SC components made of superalloys. It is now generally agreed that freckles are the product of specific fluid flow patterns, known as thermosolutal convection, originating in the interdendritic liquid during solidification. The freckling extent becomes more pronounced as the component size increases. Another factor playing an important role in freckle formation is the solidification condition of the castings, including the temperature gradient (*G*) at solidification front, the solidification velocity (*V*), the local cooling rate (*T*’), and the height of the mushy zone (*H*). These process parameters directly affect the dendrite spacing (*λ*) and the mushy zone permeability (*K*). A lower cooling rate leads to a coarser dendrite structure, reducing the resistance to the interdendritic fluid flow and increasing the likelihood of freckling [20,21]. As more and more refractory alloying elements are added to improve high-temperature mechanical properties, freckle formation in directionally solidified superalloy components has increasingly become a serious problem.

In addition to the factors mentioned above, it was found that the freckle formation is also significantly affected by the geometric shape of the superalloy castings [22,23,24,25,26,27]. On the transverse sections of the components with curved contours, freckles were exclusively found on the outward-curving surfaces with positive curvature, because the surface effect zones of the neighboring sides are overlapped, providing more favorable convection conditions. In comparison, the surfaces with negative curvature remained freckle-free, because the surface effect of the neighboring sides is divergent from each other. In the longitudinal direction, the freckle formation can be promoted by contracting contours and suppressed by expanding one, respectively. As the composition and shape of turbine blades are becoming increasingly complex, effectively reducing or eliminating freckle defects has been more challenging in manufacturing high-generation SC superalloy components.

Over the past decades, X-ray imaging has been recognized as a powerful method for solidification studies in metallic alloys. This technique enables real-time and in situ observations of thermosolutal flow during the solidification of metallic alloys, without disrupting the process [28,29]. Buoyancy-driven flow patterns have been studied in Ga-In alloys using X-ray radioscopy and further applied to investigate the formation of segregated channels, freckles, and forced convection [30,31,32]. By using a high-temperature furnace and a high-speed X-ray-sensitive camera, the development of solute flow during dendritic growth of a Ni-based superalloy CMSX-4 was directly observed [33]. It was then evidently confirmed that the solutal convection and freckle formation are unavoidable during the conventional directional solidification of superalloys.

Besides the experimental investigations, a lot of studies pertaining to freckle formation have concentrated on the development of computational models and/or the incorporation of Rayleigh-number (*Ra)*-based criteria, to predict the onset of thermosolutal convection and subsequent formation of grain defects [34,35,36,37,38]. Theoretically, *Ra* is defined as the ratio of the driving force to the resistance for buoyancy-driven convection, reflecting the competition between the thermosolutal buoyancy and the retarding friction force in the mushy zone. The Rayleigh number is influenced by the alloy compositions and the dendrite spacings, which are determined by the solidification conditions. Since it incorporates both the chemical composition and processing parameters, the Rayleigh number has become a highly promising tool for understanding the interdendritic convection phenomena. However, due to its oversimplification, the classic *Ra* criteria can still not be applied to predict the freckle formation during the industrial manufacturing process, because of the complexity of the casting shapes and the processing conditions. Recently, a revised freckle-predicting *Ra* model was proposed [39]. This model quantified the geometrical effect leading to the formation of the freckle defect and addressed the shortcomings of the existing models. Using the revised *Ra* model, the specific positions of the freckling-risk zones in complex SC castings can be determined, and the freckling tendency can be predicted.

In the present work, experiments based on industrial Ni-based superalloys were conducted, employing both the conventional upward-solidification and a novel downward-solidification techniques to investigate the feasibility of producing SC superalloy components free of freckle defects. In situ observation experiments were conducted based on a low-melting-point metal alloy (Ga-30 wt.% In) to directly observe the thermal–solutal convection behavior during solidification in both directions. Numerical simulations were also performed using the newly developed *Ra* model [39] to predict the freckle formation in both upward- and downward-solidification processes. A systematic analysis was carried out to quantify the impact of gravitational factor and solidification direction on solidification microstructure. The knowledge gained from this study proposed new approaches to address buoyancy-driven convection and the related issues in the directional solidification process of superalloys.

## 2. Experiment and Simulation

### 2.1. Solidification Experiment of a Superalloy

A commercial Ni-based SC superalloy of typical second-generation, CMSX-4, was selected for the casting experiments since it is a typical freckle-prone superalloy. The chemical composition of CMSX-4 is listed in Table 1.

**Figure 1:**
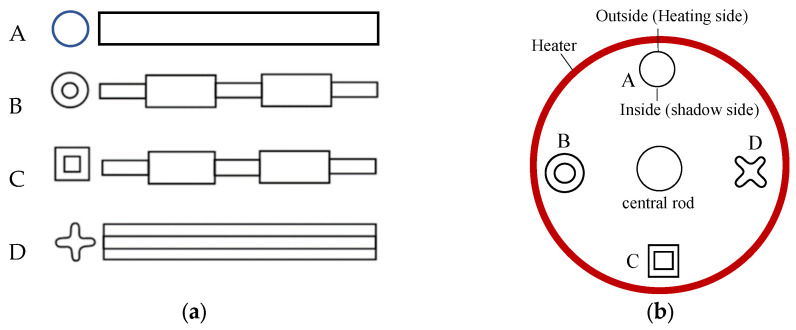
(**a**) End- and side-view of the designed casting geometries; (**b**) top-view of the casting arrangement in a cluster.

Four sample geometries, labeled A, B, C, and D, were designed for the casting experiment, with their end and side views illustrated in Figure 1a. Sample A is a simple cylindrical bar with a diameter of 15 mm. Sample B consists of some short cylindrical sections, each 30 mm in length, with diameters of 10 and 15 mm, respectively. Sample C is similar to Sample B, but the cross-sections were changed to square ones with side widths of 10 and 15 mm, respectively. The cross-section of sample D is shaped like a cross-petal, with a diagonal petal length of 15 mm.

**Figure 2:**
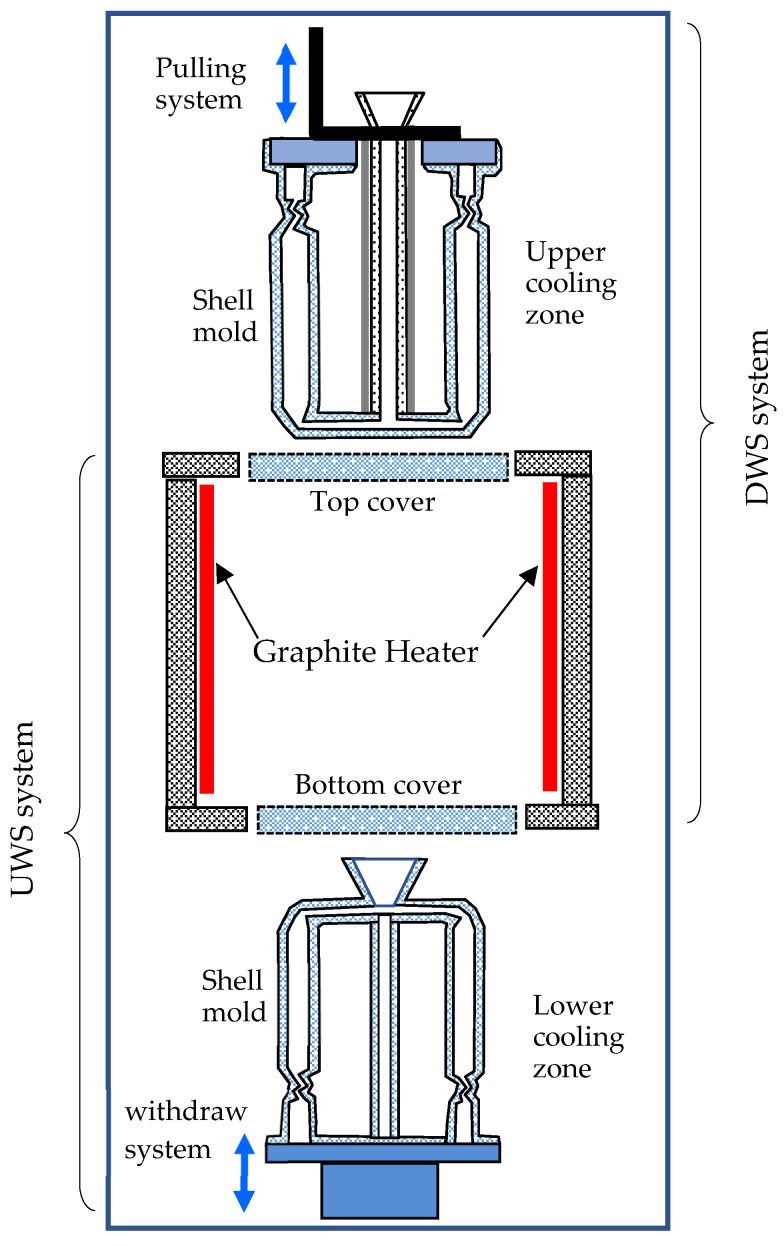
Schematic of the self-developed device for upward-solidification (UWS) and downward-solidification (DWS) experiments.

Figure 1b shows schematically the arrangement of the corresponding wax patterns of the samples in a casting cluster, which were assembled in a circular ring around a central rod on the plate. According to the lost-wax process, the wax assemblies were repeatedly coated by dipping into ceramic slurries, followed by stuccoing with ceramic sands to build a ceramic shell mold around the wax pattern, until the desired shell-wall thickness was attained. The shell molds were then de-waxed in a steam autoclave and fired in a heating furnace. It should be indicated that two different pouring systems were introduced into the shell molds (Figure 2) to perform the directional solidification experiments in two different directions, as shown below.

In the present work, a novel prototype device was developed for solidification experiments in both upward and downward directions, respectively (Figure 2). Between the upper and the lower cooling zones, a heating chamber is located in the middle, where the shell molds are preheated and poured with alloy melt.

**Figure 3:**
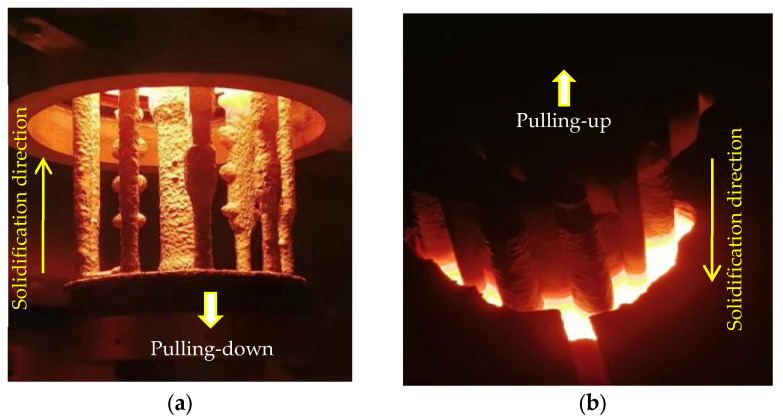
Pulling-down (**a**) and pulling-up processes (**b**) of the shell molds, resulting in upward solidification (UWS) and downward solidification (DWS), respectively.

In the first experiment, the upward solidification (UWS) system of the equipment (Figure 2) was used to lift the ceramic mold into the heating chamber, which is covered from above. After pouring the alloy melt, the mold was withdrawn downward from the heating chamber into the lower cooling chamber (Figure 3a), enabling UWS from the bottom to the top of the castings, also known as anti-gravity solidification.

In the second experiment, the downward solidification (DWS) system of the equipment (Figure 2) was used to first lower the mold into the heating chamber for preheating and pouring alloy melt and then to lift the mold upward again into the upper cooling chamber (Figure 3b). In this process, the DWS of the castings from the top to the bottom, also known as along-gravity solidification, was realized.

The UWS and DWS experiments were conducted in the same furnace heated to the same temperature of 1500 °C. The same superalloy (CMSX-4) was molten and overheated to the same pouring temperatures of 1500 °C and poured into the molds made from the same ceramic materials. In both experiments, the poured molds were directionally solidified with the same pulling rate of 1.5 mm/min but in opposite directions. In short, the same process parameters were set in both experiments to ensure the same solidification conditions, including cooling rates and thermal gradients, although they were not measured. Therefore, the structure difference between the experiments could only be attributed to the solidification direction, because the other solidification conditions were controlled to be the same.

After the casting experiments, the samples were knocked out of the ceramic mold and mechanically separated from the casting cluster. The samples were then sandblasted to remove any ceramic deposits attached to their surface and were subsequently macro-etched in a solution of 50% H_2_O_2_ and 50% HCl. Metallographic examinations were conducted to ascertain the freckle defects on the surface of the solidified samples.

### 2.2. In Situ Solidification Experiments of a Ga-30 wt.% In Alloy

Besides the directional solidification experiments of superalloy castings, in situ solidification experiments in up- and down-directions were also performed. An apparatus for in situ X-ray observation of the solidification process of a low melting point metal alloy was installed. The applied Ga-In alloy has a very low melting point, enabling the convenient conduction of in situ observation experiments with simple conditions, instead of high temperature and vacuum conditions for superalloys. More importantly, the solidification behavior of this alloy is very similar to that of superalloys, including the crystal growth in dendritic form and the density decrease in the residual liquid leading to the thermal–solutal convection. Therefore, Ga-In is a suitable material used to simulate the solidification process of superalloys. In fact, this alloy has been widely used in in situ observation experiments of upward-directional solidification, but this work is the first to use it for downward-directional solidification experiments.

The Ga-30 wt.% In alloy was prepared and filled into a windowed rectangular cell with a parallel gap of 0.5 mm. The semiconductor components were used as heater and cooler, respectively, allowing independent and precise control of the upper- and lower-zone temperatures. The solidification setup was mounted between a microfocus X-ray source and an X-ray detector. By establishing a thermal field with a positive or negative temperature gradient, upward- or downward-directional solidification was enabled. During the experiments, the dendrite growth processes accompanied by the solutal convection of the interdendritic residual liquid were in situ observed and recorded for further analysis.

### 2.3. Simulation of Superalloy Solidification

Given the urgent need for a scientific mathematical model to reasonably predict freckle defects in superalloy castings, we have developed a new version of the Ra number to characterize the tendency of solutal convection and freckle occurrence during directional solidification [39]. For the convenience of detailed explanation and discussion, this mathematical model is presented as Equation (1) in Section 4 of this paper.

To predict the freckling tendency in the castings of various shapes, as shown in Figure 1, the solidification process and temperature field evolution in the castings were calculated using the commercial software ProCAST 2019. All modeling settings are identical to the experimental conditions. Then, the relative parameters in *Ra* number (Equation (1)) at each location were evaluated. Based on the evaluated location conditions, the corresponding correction factors were applied. Finally, the Ra-number distribution in the castings during the solidification process was calculated using Equation (1) to predict the freckling tendency in the various casting shapes. Besides the geometry effect, the impact of the solidification direction on the freckling tendency was also calculated. The simulation results were compared with the experimental measurements.

## 3. Experimental Results and Discussion

### 3.1. Directional Solidification Experiments of Superalloy

**Figure 4:**
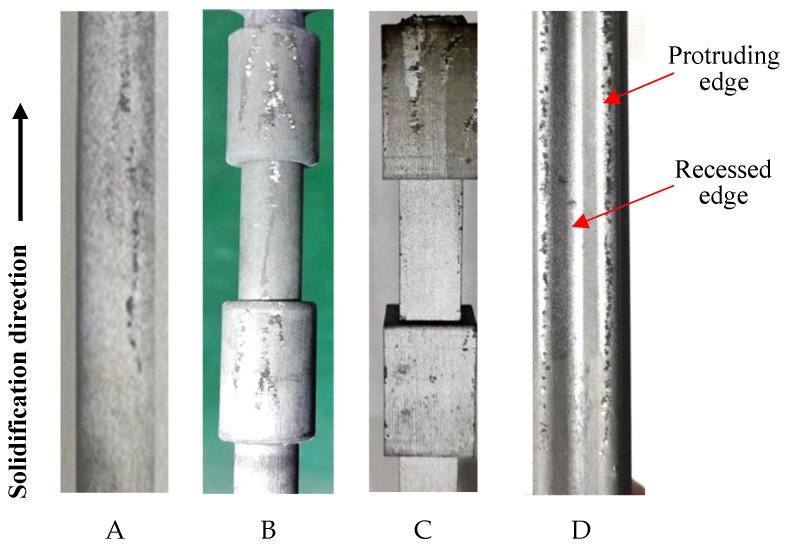
Surface photos of four samples (**A**–**D**) prepared in the UWS process.

In the first experiment, the superalloy samples were upwards-solidified (UWS process) using the conventional pulling-down method (Figure 3a). In all samples, typical vertical freckle chains can be clearly observed on the vertical surface (Figure 4). It should be noted that the freckles were found mostly on the “shadow side” of the samples (Figure 4), which faces the central rod and could not be radiated directly by the heater. On the opposite side facing the heater (heating side), significantly fewer freckles were observed. This shadow effect indicates an unsymmetrical thermal condition between the inner and outer sides of the casting cluster, which plays an important role in freckle formation.

Sample D has the shape of a cross-shaped petal, whose protruding edges on the shadow side are occupied by serious freckles, while the recessed edges with concave curvature remain freckle-free. This can be explained by the edge effect or curvature effect on the freckle formation, found in the authors’ previous work [22,23,24]. Freckles are surface defects whose formation is promoted by the surface contacting with the mold wall, revealing the so-called surface effect or wall effect. In our previous study [16], the flow permeability near the casting surface was examined to be about one order of magnitude higher than that inside the mushy zone. A boundary layer was defined to indicate the depth of the surface effect. At the convex edges having positive curvature, such as at the protruding edges in sample D (Figure 4), the surface effect zones of the neighboring sides are overlapped, providing more favorable freckling conditions. On the concave edge having negative curvature, such as the recessed edges in sample D in Figure 4, the surface effect of the neighboring sides is divergent from each other, resulting in a normally freckle-free zone. This can be called the curvature effect of the transversal geometry on the freckle formation, corresponding to the edge-effect in the polygonal sections of castings [22,23,24].

**Figure 5:**
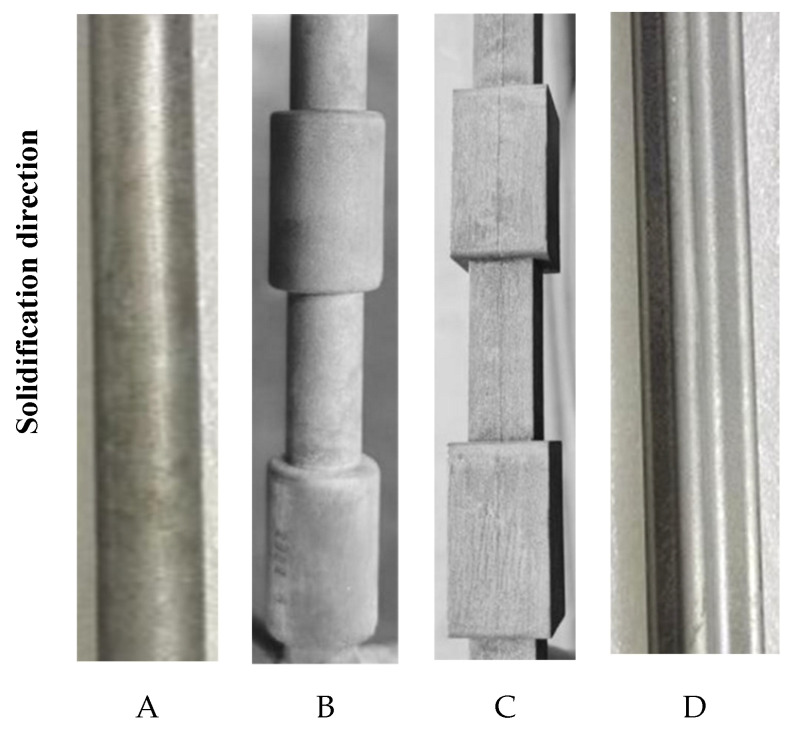
Surface photos of four samples (**A**–**D**) produced in the DWS process.

In the second experiment, the superalloy samples were downwards-solidified (DWS process) using the newly developed pulling-up method (Figure 3b). Figure 5 shows the surface photographs of the four samples on the shadow side facing the central rod, just the same position as the results in Figure 4. After careful examination, it was confirmed that none of the samples exhibited freckle defects on their surfaces, as shown in Figure 5, which is totally opposite to Figure 4. Even the protruding edges of the sample D are also freckle-free, although this position is very prone to freckle formation during the conventional Bridgman process (Figure 4). It is thereby confirmed that the downward solidification process (i.e., along gravity) has natural immunity against freckle formation in superalloy castings.

The directional solidification of superalloys is accompanied with strong segregation of alloying elements between the liquid and solid phases. The lighter elements, such as Al and Ti, have positive segregating behavior tending to enrich in residual liquid between the dendrites, while the heavier refractory elements, such as W and Re, leave the liquid and enter the solid dendrites. The density of the interdendritic liquid *ρ*_1_ in the mushy zone gradually decreases compared to the initial liquid density *ρ*_0_ ahead of the growing dendrite tip.

**Figure 6:**
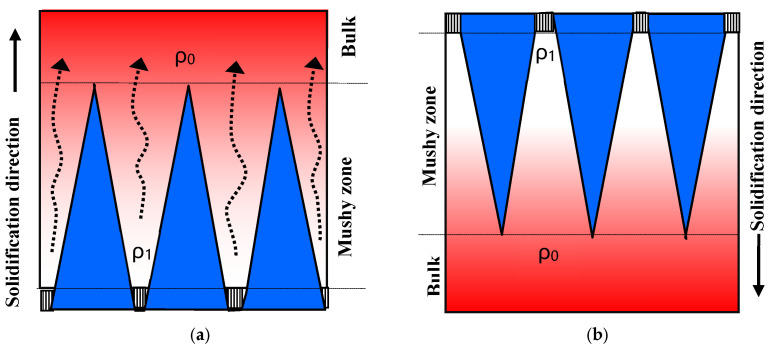
Schematic showing the influence of solidification direction on the solutal flow (as indicated by the dashed arrows): (**a**) convection during upward solidification; (**b**) stable state during downward solidification.

In the first experiment, the conventional upward solidification (UWS) was performed by pulling down the shell mold filled with alloy melt. The bulk liquid with the original density *ρ*_0_ is above the mushy zone with the residual liquid having lower density *ρ*_1_ (Figure 6a). The liquid density difference between the top and bottom of the mushy zone, is then ∆*ρ =* (*ρ_0_ − ρ_1_*) > 0. Under gravity, this below-lighter and top-heavier state in liquid is unstable, leading to thermosolutal convection between the mushy zone and the top bulk liquid. This flow pattern results in the formation of freckles consisting of broken dendrite arms.

In comparison, the freckle defects were completely eliminated in the newly developed DWS process (Figure 6b). This is because during the downward solidification process, the mushy zone with reduced liquid density *ρ*_1_, was above the bulk liquid zone with original density*ρ*_0._ The liquid density difference between the top and bottom of the mushy zone, is then *∆ρ* = (*ρ_1_ − ρ_0_*) < 0, forming an absolutely stable state in the below-heavier and top-lighter zone. In this case, the gravitational effect was transformed from a destabilizing factor to a stabilizing one. Due to the absence of a driving force for the solutal convection, the freckle defects could be completely avoided.

In order to most convincingly confirm the effectiveness of the UWS method in eliminating freckle defect, the experiments were conducted under extreme conditions to stimulate freckles. For this purpose, very low pulling velocity (*V* = 1.5 mm/min) and very low heating temperature (*T* = 1500 °C) were applied, so that severe freckles were formed in the conventional UWS process. Under the same serious condition, however, freckle-free castings were obtained by using the new UWS method, providing the most powerful evidence for the effectiveness of UWS method.

### 3.2. Result of In Situ Solidification Experiments

In this work, in situ observation experiments of bidirectional solidification were carried out using thin-sheet samples of the low-melting-point alloy Ga-30 wt.% In. This alloy exhibits a similar solidification behavior to that of superalloys, such as the dendritic growth and the density reversion in the mushy zone. Specifically speaking, during the solidification process, the residual interdendritic liquid is enriched in Ga elements (ρ_Ga_ = 5.91 g/cm^3^) and depleted in In elements (ρ_In_ = 7.3 g/cm^3^). As a result, the residual liquid between the dendrites in the mushy zone becomes lighter than the original melt.

**Figure 7:**
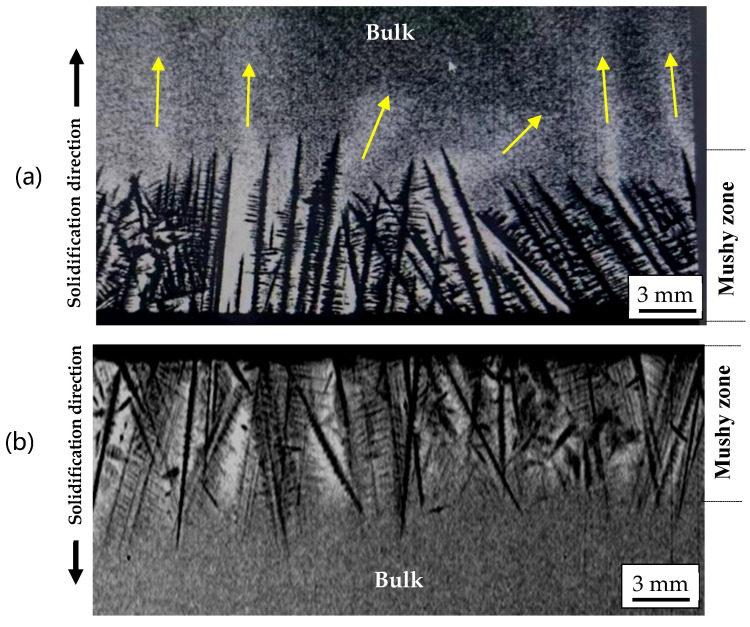
In situ observation of the upward-solidification with strong plume flow as indicated by arrows (**a**) and the downward solidification without convection (**b**), the yellow arrows represent the direction of solute flow.

In the first experiment, the upward-solidification experiment was performed and in situ observed. As shown in Figure 6a, the dendrites were growing upwards, similar to the solidification phenomena during the conventional directional solidification. Compared with the bulk liquid zone at the top, the residual liquid between the dendrites appears brighter due to its lower density, which is attributed to the enrichment of the lighter Ga element. Under gravity, the interdendritic lighter liquid was in an unstable state. The lighter liquid located at the lower part of the mushy zone flowed upwards, inducing the formation of a plume flow. The flow direction of the liquid within the plume is indicated by the arrows in Figure 7a. This phenomenon has also been confirmed in in situ observation experiments of superalloys under high-temperature and vacuum conditions [33].

In the second experiment, a downward-solidification of the Ga–In alloy was performed, where the mushy zone with lighter residual liquid was over the bulk liquid (Figure 7b). In this case, the lighter liquid remained at the top between the dendrites, and no plume flow developed from the mushy zone downwards into the bulk liquid below. This could be attributed to the absolute stable state with the lighter liquid at the top and the heavier one at the bottom. The density difference between the interdendritic liquid and bulk one becomes a suppressing force. This is in contrast to the conventional Bridgman process, where it acts as a driving force for the onset of solutal convection. This in situ observation experiment directly provides strong evidence for the importance of solidification direction on the solidification behavior. It clearly shows that by simply changing the solidification direction from against gravity to along gravity, the solutal convection and subsequent freckle formation could be completely avoided. This was experimentally confirmed in Figure 4 and Figure 5, offering a new approach to produce freckle-free SC superalloy blades.

## 4. Results and Discussion of Simulation Work

Freckle is a typical structural defect formed in directionally solidified superalloy castings, but its formation mechanism has not yet been reasonably explained. In addition to a large amount of experimental work, mathematical modeling, and numerical simulation methods are also commonly used to describe freckle formation. Since the freckles are believed to originate from the liquid flow between directionally growing dendrites, various Rayleigh-number (*Ra*) have been developed to characterize the liquid instability in the mushy zone and to predict the onset of freckles [34,35,36,37,38]. However, these *Ra* criteria were derived under simple idealized conditions and were generally expressed as functions of alloy composition and solidification conditions, thereby cannot be applied to industrial casting production with complex conditions. For example, the traditional *Ra* numbers do not account for important factors such as the wall effect of actual liquid flow and the effect of casting shape. As a result, the predicted freckling locations often do not align with the experimental results.

Recently, we have developed a new version of the *Ra* number [39] to characterize the tendency of solutal convection and freckle occurrence during directional solidification of complex castings. This new *Ra* number can be briefly described in the following equation:

(1) $$Ra=(\frac{\Delta \rho }{\rho_{0}}\frac{gK_{0}H}{\alpha \nu }{)(F}_{f}F_{i}F_{d})$$

The factor in the first bracket of this equation is the expression for the traditional *Ra* number, which represents the ratio of the driving force to the viscous in the mushy zone. In this equation, *ρ*_0_ is the initial density of the bulk liquid, Δ*ρ* is the density difference between the bulk liquid and the liquid in mushy zone, *H* and *K*_0_ are width and flow permeability (*K*_0_ = 6.0 × 10^−4^ λ^2^, λ is the primary dendrite arm spacing) of the mushy zone, *α* is the thermal diffusivity, *ν* is the kinematic viscosity, and *g* is the gravitational acceleration.

The factor in the second bracket of Equation (1) introduces several correction factors accounting for the influence of shape effects, which were concretely described in reference [39]. Among them, the correction factor *F_f_* reflects the influence of the casting’s external profile on the liquid flow. A physical model with different flow paths was established based on the internal mushy zone and along the mold wall, considering the overlapping effect of the wall effect at the corners. This enables the model to represent the surface-effect and edge-effect of freckles. The factor *F_i_* in Equation (1) is a correction factor for the variation in flow intensity with changes in the longitudinal shape of the castings, which characterizes the volume scale supporting liquid flow in the mushy zone. Every expansion of the casting profile indicates an outward extension of the surface, which, due to the reduced support volume from below, weakens liquid convection and freckle formation on the surface. On the other hand, each inward contraction of the casting surface indicates the expansion of the convection-supporting region from below, making freckles more likely to occur. Therefore, *F_i_* represents the correction effect of shape contraction or expansion in the solidification direction. *F_d_* in Equation (1) represents the correction factor for the change in mushy zone width influenced by the shadow effect. This model can be used to calculate the *Ra* number at every position of SC castings. It realized the evaluation of the freckling tendency of the whole casting and the location of the specific positions of the freckling risk zones.

In addition, the factor Δ*ρ* in Equation (1) represents the density difference between the liquid at the top and that at the bottom of the mush zone. The sign of factor Δ*ρ* is decided by the solidification direction. In conventional upward directional solidification (Figure 6a), the interdendritic liquid becomes increasingly lighter, reaching the minimum *ρ*_1_ at the bottom of the mushy zone. This results in Δ*ρ =* (*ρ*_0_ − *ρ*_1_) > 0, i.e., *Ra* > 0, indicating a positive driving force for solutal convection and thus a high risk of freckle formation. In the newly developed downward solidification (Figure 6b), however, the density factor becomes negative, *Δρ =* (*ρ*_1_ − *ρ*_0_) < 0, i.e., *Ra* < 0, indicating a negative driving force for convection. In this case, the role of gravity changes from being the driving force for the solutal convection to a suppressing force, thereby achieving a stable liquid state and freckle-free solidification.

In order to predict the freckling tendency in the various casting shapes that are shown in Figure 1, the *Ra* number during the solidification process was calculated for each sample. Firstly, the solidification process and temperature field evolution in the castings were calculated using ProCAST 2019. Then, the relative parameters in *Ra* number (Equation (1)) at each location were evaluated, including the height of the mushy zone *H*, the density difference Δ*ρ* between the top and the bottom of the mushy zone, and the flow permeability *K*_0_ of the mushy zone. Based on the location conditions, the corresponding correction factors were applied. Finally, the *Ra-*number distribution in the castings during the solidification process was calculated using Equation (1).

**Figure 8:**
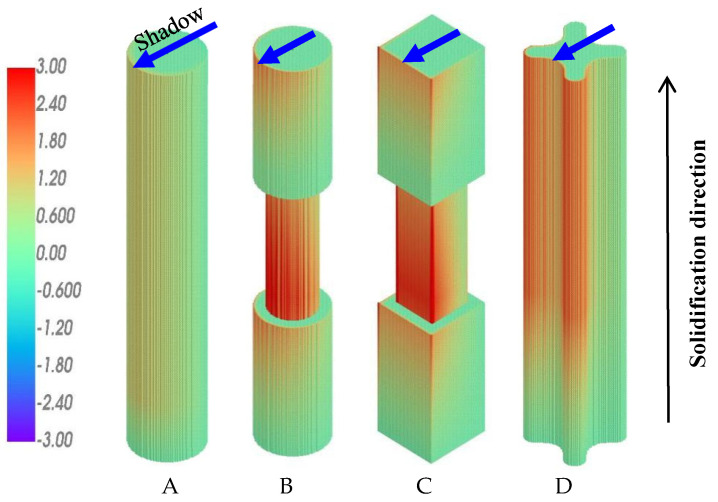
Simulation result for *Ra* distribution in the samples (**A**–**D**) during conventional UWS process.

The calculated *Ra-*distribution for these four samples during the conventional upward solidification (UWS) process is shown in Figure 8. On the shadow side facing the central rod (indicated by arrows), the calculated *Ra* number is significantly higher than that on the heating side facing the heater. This is in good agreement with the experimental results shown in Figure 4, where all the freckles appear on the shadow side of the castings.

In castings A and D, which have no variation in cross-sections along the longitudinal direction, the calculated *Ra*-values in the upper part are greater than those in the lower part of the castings. This is attributed to the corresponding change in the solidification condition. As the casting system moves downward from the top hot chamber to the lower cold chamber, the temperature gradient and cooling rate decrease gradually, leading to the coarsening of dendrite structures and increased freckling tendency. Casting D has a cruciform cross-section, where the *Ra* number at the edges is significantly higher than that on the smooth surface of cylindrical casting A. This simulation result confirms the influence of the edge effect on freckle formation and is consistent with the experimental results (Figure 4).

In the stepped samples B and C, the cross-section suddenly decreases and increases along the solidification direction, leading to an increase and decrease in the *Ra* number, respectively. As a result, the step effect on the freckle formation, i.e., the promoting effect of contour contraction and suppressing effect of contour expansion, is also predicted by the current simulations in this work. This effect has been confirmed in the previous and present experimental works.

**Figure 9:**
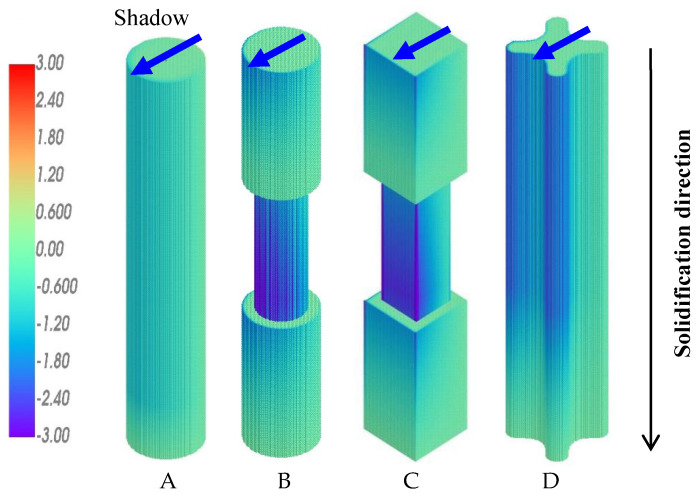
Simulation result for *Ra* distribution in samples (**A**–**D**) during the newly developed DWS process.

As mentioned above, the sign of the density difference between the top and bottom of the mushy zone, ∆*ρ*, is related to the solidification direction. The simulation results shown in Figure 8 were calculated under the condition of upward solidification against gravity. The bulk liquid is above the upward-moving mushy zone, so the density difference between the top and bottom of the mushy zone, ∆*ρ*, is greater than 0. The result of ∆*ρ* > 0, and thereby *Ra* > 0, indicates a positive driving force for the liquid flow and freckle formation, as shown in Figure 6a. However, for the downward solidification process, the dendrites grow downward into the bulk liquid below the mushy zone. The density difference *∆ρ* between the top and bottom of the mushy zone, i.e., between the dendrite rood and dendrite tip, is less than 0, leading to *Ra <* 0 for all positions in all castings, as shown in Figure 9. In this case, the driving force exerted by gravity on the liquid flow becomes negative, resulting in a convection-free and, consequently, a freckle-free solidification. This prediction has also been confirmed by the corresponding experimental results in Figure 5 and explained by the schematic illustration in Figure 6b.

It is worth emphasizing that the sign of ∆*ρ* and *Ra* in Equation (1) is determined by the solidification direction. In the conventional upward-solidification process against gravity, the positive sign of ∆*ρ* and *Ra* means a positive buoyant force to drive liquid convection, leading to an unstable state with an increased risk of freckle formation. In the downward-solidification process, as applied in the present work, however, the sign of ∆*ρ* and *Ra* in Equation (1) becomes negative, indicating a negative driving force for liquid convection, which suppresses liquid convection and reduces the risk of freckle formation. In other words, the density reduction in the mushy zone above the bulk liquid is a suppressing factor, instead of a promoting factor, for solutal convection. The higher the *Ra*-value, the more stable the liquid, due to its negative sign. In this case, regardless of all other conditions, such as alloy composition, solidification rate, and casting shape, an absolutely stable state is established for solidification free of convection and freckles.

## 5. Conclusions

(1) During the conventional upward solidification of superalloy CMSX-4, severe freckles occurred on the castings of various shapes. In contrast, freckle-free castings were obtained simply by changing the solidification direction from upward to downward.
(2) To visually verify the effect of solidification direction, in situ observation experiments of bidirectional solidification were performed using a Ga-In alloy. In the upward-solidification process, strong solutal convection was visually observed due to the decrease in the interdendritic liquid density. On the other hand, a convection-free state was achieved during downward solidification, because of the stable state under the top-light and bottom-heavy conditions.
(3) A new Rayleigh-number model was successfully applied to characterize the freckle features in superalloy cluster castings. When the solidification direction changed from upward to downward, the driving force for solutal convection was predicted to change from positive to negative. In this case, an absolutely stable state in the melt was established, leading to the complete avoidance of freckle formation in single-crystal superalloy castings.
(4) Based on the result of this work, the downward solidification process should be further applied to the production research of large superalloy components, which is even more important because they are more prone to freckle defects and cannot be avoided by conventional methods.

## Figures and Tables

**Table 1:** Nominal composition of superalloy CMSX-4 (wt.%).

Cr	Co	W	Mo	Al	Ti	Ta	Re	Hf	Ni
6.5	9.0	6.0	0.6	5.6	1.0	6.5	3.0	0.1	Bal.

## Data Availability

The original contributions presented in this study are included in the article. Further inquiries can be directed to the corresponding author.
